# *Mcph1*-Deficient Mice Reveal a Role for MCPH1 in Otitis Media

**DOI:** 10.1371/journal.pone.0058156

**Published:** 2013-03-13

**Authors:** Jing Chen, Neil Ingham, Simon Clare, Claire Raisen, Valerie E. Vancollie, Ozama Ismail, Rebecca E. McIntyre, Stephen H. Tsang, Vinit B. Mahajan, Gordon Dougan, David J. Adams, Jacqueline K. White, Karen P. Steel

**Affiliations:** 1 Wellcome Trust Sanger Institute, Hinxton, Cambridgeshire, United Kingdom; 2 Edward S. Harkness Eye Institute, Columbia University, New York, New York, United States of America; 3 Omics Laboratory, Department of Ophthalmology and Visual Sciences, University of Iowa, Iowa City, Iowa, United States of America; VIB & Katholieke Universiteit Leuven, Belgium

## Abstract

Otitis media is a common reason for hearing loss, especially in children. Otitis media is a multifactorial disease and environmental factors, anatomic dysmorphology and genetic predisposition can all contribute to its pathogenesis. However, the reasons for the variable susceptibility to otitis media are elusive. *MCPH1* mutations cause primary microcephaly in humans. So far, no hearing impairment has been reported either in the *MCPH1* patients or mouse models with *Mcph1* deficiency. In this study, *Mcph1*-deficient (*Mcph1^tm1a^* ^/*tm1a*^) mice were produced using embryonic stem cells with a targeted mutation by the Sanger Institute's Mouse Genetics Project. Auditory brainstem response measurements revealed that *Mcph1^tm1a^* ^/*tm1a*^ mice had mild to moderate hearing impairment with around 70% penetrance. We found otitis media with effusion in the hearing-impaired *Mcph1^tm1a^* ^/*tm1a*^ mice by anatomic and histological examinations. Expression of Mcph1 in the epithelial cells of middle ear cavities supported its involvement in the development of otitis media. Other defects of *Mcph1^tm1a^* ^/*tm1a*^ mice included small skull sizes, increased micronuclei in red blood cells, increased B cells and ocular abnormalities. These findings not only recapitulated the defects found in other *Mcph1*-deficient mice or *MCPH1* patients, but also revealed an unexpected phenotype, otitis media with hearing impairment, which suggests *Mcph1* is a new gene underlying genetic predisposition to otitis media.

## Introduction

Otitis media (OM), inflammation of the middle ear, is the most common cause of hearing impairment in children. As a multifactorial disease, the pathogenesis of OM is complicated. Based on previous research, many factors are thought to contribute to the development and persistence of OM including: environmental factors such as smoking and type of child care; anatomical dysmorphology; Eustachian-tube function; adaptive and innate immune system function; viral and bacterial load; and genetic predisposition. However, the mechanisms underlying OM are still elusive. Heritability estimated from twin studies [1], [2] and linkage analysis [3] indicates a strong genetic component is involved in OM. Outcomes vary in different patients with similar symptoms after standardised treatment, also suggesting differences in their underlying pathophysiology that may have a genetic component. Clinical studies of OM are limited by the wide range of environmental elements involved. Mice, however, can be bred in a controlled environment, minimising the variation arising from the environment, making it a useful model for building an understanding of the genetic pathways and mechanisms underlying OM. Due to our very limited knowledge of the genetic etiology of OM in humans, it is hard to anticipate which genes may contribute to this disorder. As summarised in a recent review [4], mouse models involving disruption of genes functioning as transcription factors, and genes involved in apoptosis, the immune system, ciliary function and in mucopolysaccharidoses revealed that many pathways and processes can contribute to the development of OM.

In the present study, microcephalin 1 (*Mcph1*)-deficient (*Mcph1^tm1a^* ^/*tm1a*^) mice were found to exhibit hearing impairment as a part of the Sanger Institute's Mouse Genetics Project (MGP). The MGP uses the Knockout Mouse Project and the European Conditional Mouse Mutagenesis Program (KOMP/EUCOMM) resource of over 17,000 genes targeted in ES cells [5] and aims to generate and screen the phenotype of mutants for 160 genes per year. The hearing screening uses the Auditory Brainstem Response (ABR) test at 14 weeks of age and is part of the standardised battery of primary phenotypic tests.

*MCPH1* mutations cause primary microcephaly in humans characterized by a markedly reduced brain size and mental retardation [6], [7]. The *MCPH1* gene (also known as *BRIT1*, BRCT-repeat inhibitor of hTERT expression) encodes MCPH1, which contained three BRCT domains: one in its N terminus and two in its C terminus. BRCT domains have been found predominantly in proteins involved in cell cycle checkpoint functions and in proteins involved in the DNA damage response [8], [9]. Because the MCPH1 protein contains three BRCT domains, previous studies focused on and implicated MCPH1 in DNA damage response [8], DNA damage regulation in the ATM/ATR pathways [10], co-ordination of the regulation of Cdc25A and Cdk1–cyclin B1 activity and thus, in regulation of mitotic entry [11], and homologous recombination repair through the Condensin II complex [12]. MCPH1 also has a role in coupling the centrosome cycle with mitosis, which is required for precise mitotic spindle orientation and regulation of progenitor cell division to maintain brain size. Neuroepithelial cells have apical-basal polarity, and the switch from proliferative, symmetric to neurogenic, asymmetric division is controlled by the orientation of the spindle pole during mitotic division. Primary microcephaly is caused by mutations of centrosomal proteins and is thought to arise from an increase in asymmetric divisions that reduces the size of the neural progenitor pool available for future brain growth [13]. Finally, MCPH1 has been proposed to function as a tumor suppressor gene that contributes to both cancer initiation and cancer progression in a variety of cancer lineages [10].

The broad phenotypic screening of the MGP revealed that *Mcph1^tm1a^* ^/*tm1a*^ mouse mutants not only had some expected features such as small skull size and increased micronuclei reflecting genome instability, but also showed some unexpected phenotypes including susceptibility to OM implicating MCPH1 in genetic predisposition to OM. This finding implicates a new molecule in the pathogenesis of OM that is relevant to understanding the underlying mechanisms irrespective of the initial trigger for OM.

## Materials and Methods

All mouse breeding and investigation was carried out with authorization of the UK Home Office. Mice were killed by cervical dislocation and decapitation. All efforts were made to minimize suffering.

### Production of *Mcph1^tm1a/tm1a^* mice

**Figure 1:**
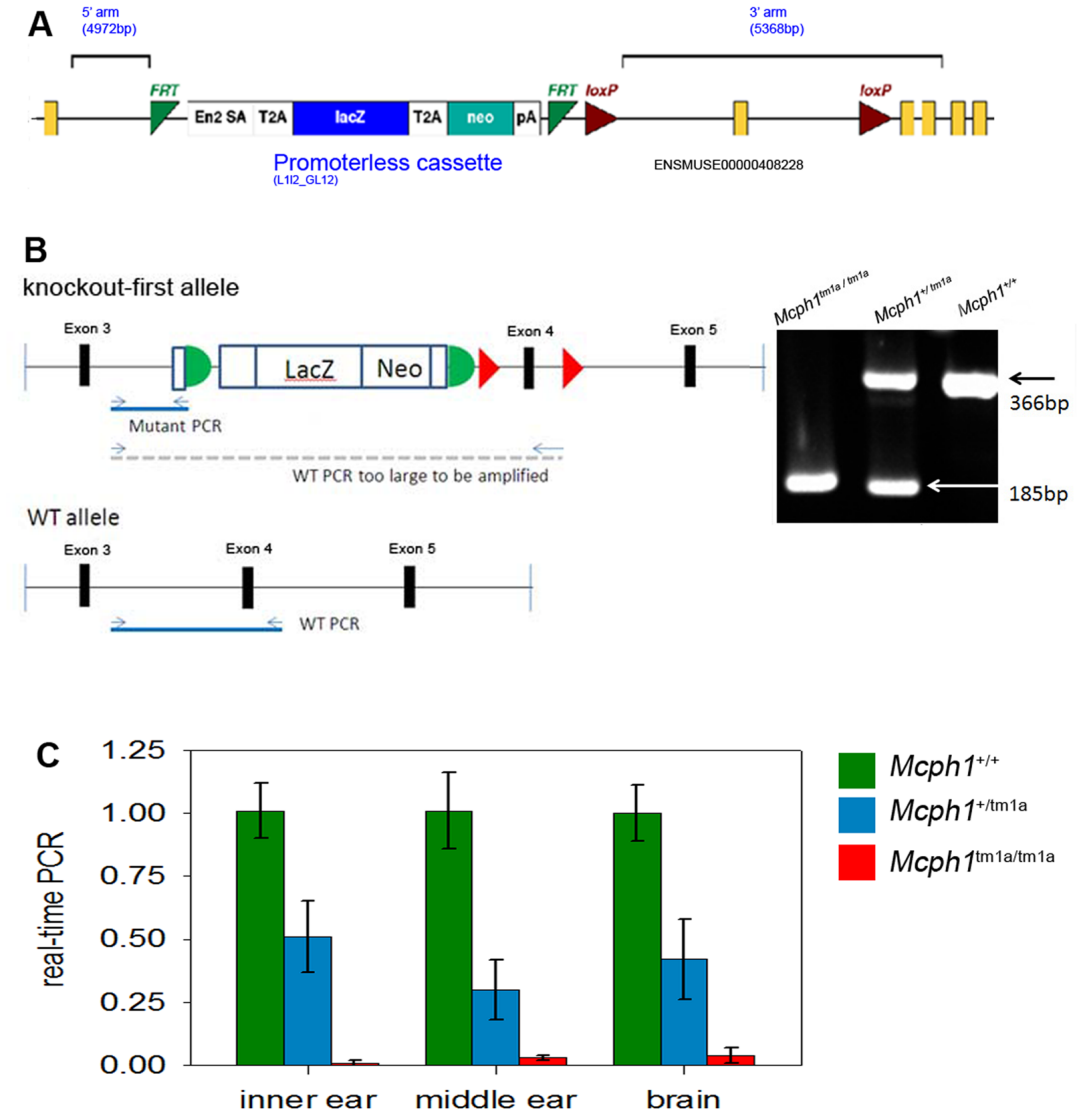
Production of *Mcph1*-deficienct (*Mcph1^tm1a/tm1a^*) mice. (A) Schematic of knockout strategy for *Mcph1* gene based on knockout-first design. A promoterless cassette including *LacZ* and *neo* genes was inserted in the third intron of *Mcph1* gene flanked by FRT sites. *LoxP* sites flank the critical exon (exon4 of *Mcph1* gene in knockout-first design). See http://www.knockoutmouse.org/martsearch/project/41705 for more details. (B) Short range PCR for genotyping. Wild type allele produces one band of 366bp. Due to the insertion of the cassette, primers designed for the wild type allele do not have product for the mutant allele using the short range PCR (illustrated as the left panel, schematic illustration is not in scale). The homozygous allele produces only one band of 185 bp. The heterozygotes produce two bands of 185 bp and 366 bp. (C) Quantitative real-time PCR showed largely reduced transcript of *Mcph1* in *Mcph1^tm1a^* ^/*tm1a*^ (n = 3) mice compared to wild type mice (n = 3) and the residual levels vary in different organs.

*Mcph1*-deficient (*Mcph1^tm1a(EUCOMM)Wtsi^*, abbreviated to *Mcph1^tm1a^* in this report) mice carry a knockout-first allele [5], in which a promoterless cassette including *LacZ* and *neo* genes were inserted in intron 3–4 of the *Mcph1* gene (Figure 1A). In the knockout-first allele design, the knockout is obtained by introduction of a splice acceptor/reporter cassette with a strong polyA site into an endogenous intron upstream of a critical exon. By computer analysis based on defined criteria (http://www.knockoutmouse.org/kb/entry/102/), exon 4 of *Mcph1* was chosen as the critical exon. The vectors containing the *Mcph1* knockout-first allele were electroporated into embryonic stem cells (JM8F6) derived from C57BL/6N mice. Targeted embryonic stem cell lines were selected using neomycin and screened by long range PCR after homologous recombination. The presence of the *LoxP* site was confirmed by sequencing. Correct integration of the 5′ arm and 3′ arm was confirmed by long range PCR using a universal primer and two genome-specific primers, and the subsequent PCR amplicon was verified by sequencing. The positive stem cells were injected into host mouse blastocysts and were used to generate chimeras containing the targeted allele. Male chimeras with 80–90% of targeted cells were bred with C57BL/6Brd-*Tyr^c-Brd^* females and germ line transmission of the *Mcph1* knockout-first allele was confirmed by a series of genotyping PCR analyses (http://www.knockoutmouse.org/kb/25/) using the mouse tissue DNA as template. These heterozygous mice were inter-crossed to expand the colony. The mice were maintained in individually-ventilated cages at a standard temperature and humidity and in specific pathogen-free conditions on the mixed C57BL/6N and C57BL/6Brd-*Tyr^c-Brd^* genetic background. To genotype animals (Figure 1B), DNA was extracted from the tissue of ear-clips and used as the template for short range PCR using the forward primer for the wild type allele: TGGAGTTTGGAGGGTGCTTC, and the reverse primer: CTTGGGGAATGAGGAAGGTG. The mutant allele shares the same forward primer with wild type, and the reverse primer: TCGTGGTATCGTTATGCGCC.

### Reverse transcription PCR and real-time quantitative PCR

RNA was isolated from the tissues of middle ear, inner ear and forebrain. Littermates were used (wild type mice, n = 3; heterozygous mice, n = 3; homozygous mice, n = 3, at postnatal day 3). Total RNA was isolated with QIAshredder columns (QIAgen, cat. no. 79654) and RNAeasy mini kit (QIAgen, cat. no. 74104). cDNA was synthesized with normalization of the same original amount of RNA using oligo dT and SuperScrip II (Invitrogen). Primers were designed to amplify part of exon 9–13 (forward primer: AGAAGAAAAGCCAACGAGAACATT, reverse primer: CTGAGGGGCTGGGCTGACTTG) and exon 14 (3′ UTR, forward primer: CGTGCCATCATCAGGTCAATCA, reverse primer: GGGGCGAGGAGCAAGTCTGTA). Real-time PCR was performed in quadruplicate for each sample using the probe (Applied Biosystem, Mm00557495_m1, covering exon 3–4 boundary) in an ABI Prism 7000 (Applied Biosystem). Hypoxanthine-guanine phosphoribosyltransferase (Hprt) was amplified simultaneously (Applied Biosystem, Mm01318747_g1) as the internal reference. The relative quantity of *Mcph1* RNA was calculated using 2^−ΔΔCt^ method [14].

### Auditory Brainstem Response

Mice were anaesthetised by ketamine hydrochloride (100 mg/Kg, Ketaset®, Fort Dodge Animal Health) and xylazine hydrochloride (10 mg/Kg, Rompun®, Bayer Animal Health) and subcutaneous needle electrodes were inserted on the vertex (active), and over the left (reference) and right (ground) bullae. A calibrated sound system was used to deliver free-field click (0.01 ms duration) and tone pip (various frequencies from 6–30 or 6–42 kHz of 5 ms duration, 1 ms rise/fall time) stimuli at a range of intensity levels in 3 or 5 dB steps. Averaged responses to 256 stimuli, presented at 42.2 per second, were analysed and thresholds established as the lowest sound intensity giving a visually detectable ABR response. The peak-peak amplitude of wave 1 of click-evoked ABRs (P1-N1 amplitude) was measured and plotted as a function of sound level above threshold to produce input-output functions (IOFs). The IOF slope from 0–35 dB above threshold was calculated since 0–35 dB above threshold covered the more linear range before the function begins to flatten towards a plateau and also to ensure that a similar dB SL (sensation level) range was covered in both wild type and *Mcph1^tm1a/tm1a^* mice. In more severely impaired mutants, it was not possible to record in the higher ranges of dB SL. A *t*-test was used to compare the slopes.

### Anatomy and histology of temporal bone

Mice used for recurrent ABR measurements were sacrificed after the last measurement and the anatomy of their middle ears was examined. Briefly, the external ear canals, tympanic membranes, ossicles and middle ear cavities were carefully dissected, examined and imaged. The inner ears were dissected out, fixed in 4% paraformaldehyde and examined following inner ear clearing in glycerol [15]. Skulls from 17 mice (wild type mice, n = 4 at 4–5 weeks old; heterozygous mice, n = 2 at 5 weeks old, n = 1 at 60 weeks old; homozygous mice, n = 7 at 4–6 weeks old, n = 3 at 59–61 weeks old) were collected after the ABR measurement and fixed in 10% buffered formalin, decalcified in 10% EDTA until the tissues were soft, dehydrated and embedded in paraffin. Eight-micrometer-thick sagittal sections were obtained before hematoxylin-eosin staining.

### Scanning Electron Microscopy

Inner ears of six mice at postnatal day 4 (three mice each of homozygote and wild type) were fixed by 2.5% glutaraldehyde in 0.1 M sodium cacodylate buffer with 3 mM CaCl_2_ at room temperature for 3 hours. Cochleae were finely dissected in PBS. This was followed by further processing by an osmium-thiocarbohydrazide-osmium (OTOTO) method [16]. The samples were dehydrated in increasing concentration of ethanol, critical-point dried, mounted and examined under a HITACHI S-4800 scanning electron microscope.

### Immunohistochemistry

Immunohistochemistry staining was performed using the Ventana Discovery machine (Roche) and reagents according to the manufacturer's instructions. Paraffin sections from wild type mice at postnatal day 7 (n = 3) and 4 weeks old (n = 5) were obtained as described above. The expression of Mcph1 was detected by using Mcph1 antibody (Abcam: ab2612, 1∶3000).

### X-ray assay and brain weight measurement

Digital X-ray images are acquired using a Faxitron system MX20 (Faxitron X-ray corporation) in 14-week old mice (wild type mice, n = 7M & 7F; homozygous mice, n = 6M & 7F). Mice were anaesthetised and up to five standard images were taken for each mouse. The entire skeleton morphology was assessed using a standardised protocol capturing 41 parameters. To extract the brains, a vertical incision was made through the scalp and both sides were peeled back. Then a vertical cut was made through the midline of the skull using a pair of scissors and the skull was peeled off with a pair of fine forceps. Brains were then removed using a fine spatula and weighed on a Sartorius TE212, accurate to two decimal places.

### Micronucleus assay

The prevalence of micronucleated normochromatic erythrocytes (MN-NCE) was determined using a flow cytometric assay of micronucleus formation [17]. Flow cytometry analysis was performed as described previously [17].

### Pathogen challenge experiment

*Salmonella enterica* serovar Typhimurium M525 [18] and Citrobacter rodentium [19] were grown at 37°C as stationary overnight cultures in Luria-Bertani (LB) broth (Difco) containing ampicillin (100 µg/mL Sigma) or naladixic acid (100 µg/mL Sigma) respectively. For oral inoculation, overnight cultures were centrifuged at 15,000× g for 10 min and resuspended in the original volume in phosphate-buffered saline (PBS). Bacteria were administered with a blunt-tipped gavage needle. For intravenous inoculation, overnight cultures were diluted in PBS and injected into the lateral tail vein. Inocula were cultured retrospectively on LB agar to determine the administered dose. At regular time points post-infection, faecal samples from individual mice were collected in separate sterile tubes. Faecal samples were weighed and for every 0.01 g of faeces 100 µL of sterile PBS was added. Faecal samples were homogenised on a vortex and serially diluted. The number of viable bacteria was determined by viable count on LB agar containing naladixic acid (100 µg/mL Sigma). At selected time points post-infection, mice were killed by cervical dislocation and surface-sterilized with 70% ethanol. Livers and spleens were removed aseptically into bags and weighed. Organs were homogenised in 5 mL of sterile double distilled water using a Seward Stomacher 80 (Seward, London UK) for 2 minutes at high speed. The number of viable bacteria in organ homogenates was determined by serial dilution and viable count on LB agar supplemented with an appropriate antibiotic.

### Peripheral Blood Lymphocyte profile

Flow analysis was performed on heparinised blood collected from 16-week old mice (wild type mice, n = 35F & 29M; homozygous mice, n = 7F & 7M). The following parameters were analysed: percentages of total T cells, CD4+ and CD8+ T cells, NKT cells, NK cells, B cells, Granulocytes and Monocytes are presented relative to the total CD45+ WBC population. Percentages of memory CD4 and CD4+CD25+ Treg cells are presented relative to the total CD4+ T cell population. Percentages of memory CD8 and mature IgD+ B cells are presented relative to the total CD8+ T cell and B cell populations respectively. All samples are analysed on a BD LSR II.

### Antibody level assay

Recipient mice (wild type mice, n = 2F & 4M; homozygous mice, n = 2F & 4M) were immunized by intranasal inhalation of 30 µl PBS containing 10 mg TetC (gift from Omar Qazi, Imperial College London) combined with 1 mg heat-labile toxin of *Escherichia coli* (gift of Rino Rappuoli, Chiron) adjuvant. Mice were boosted on days 7 and 21. Serum samples were collected on days 28. Detection of TetC-specific antibodies from sera was performed by ELISA. For the measurements of antibody levels, mouse blood was collected by cardiac puncture, and serum was prepared and stored at −20°C. For antigen specific antibody measurements in mouse serum, Nunc MaxiSorp plates were coated overnight at 4°C with 2 mg/ml tetanus toxin fragment C recombinant protein (TetC) in 0.1 M Na2HPO4 (pH 9.0), blocked with 3% (w/v) BSA in PBS for 1 h, and incubated with 5-fold serial dilutions of mouse serum in PBS with 1% BSA for 1 h. The plates were developed with anti-mouse Ig HRP-conjugated Abs (Dako), followed by o-phenylenediamine substrate tablets (Sigma-Aldrich) dissolved in water. Absorbance was read at 490 nm and optical densities represented as titres.

### Ocular assessment and histological examination

Mice underwent ophthalmic screening at 15 weeks of age. They were assessed for gross morphological changes to the eye using a slit lamp (Zeiss SL130) and ophthalmoscope (Heine Omega 500). The eye was examined both undilated and dilated (tropicamide). Images using the slit lamp were collected using a Leica DFC420 camera. The mice were culled under terminal anaesthesia followed by cervical dislocation and six eyes from 3 male homozygous mutants and 3 wild type mice were removed and fixed. Pupil-optic nerve sections were processed with hematoxylin and eosin, and standard images were captured under light microscopy for review [20].

### Haematology of peripheral blood

Blood was collected at 16 weeks of age into ETDA microvette tubes (Startedt) and analyzed on an analyzer (SciIVet Animal Blood Counter) with ten parameters tested.

### Clinical chemistry

16-week-old mice were terminally anaesthetized and blood was collected from the retro-orbital sinus into lithium-heparin tubes. The plasma was immediately analyzed on an Olympus AU400 Analyzer with 28 parameters tested.

### Glucose tolerance

We assessed glucose tolerance in mice fed on a high-fat diet (Western RD, 829100, Special Diets Services) from 4 weeks of age until 13 weeks of age. At 13 weeks, mice were fasted overnight before a blood sample was taken and glucose was measured using an Accu-Chek Aviva (Roche). A bolus of glucose (2 mg/g) was administered intraperitoneally and blood glucose concentration from the tail tip was measured using Accu-Chek Aviva (Roche) after 15, 30, 60 and 120 min.

## Results

### Production of *Mcph1*-deficient mice

Targeted ES cells from the EUCOMM resource were used by the MGP to generate the *Mcph1* mutant on a C57BL/6 genetic background. The mutant allele is designated *Mcph1^tm1a(EUCOMM)Wtsi^*, and abbreviated to *Mcph1^tm1a^* in this study. The design of the *Mcph1^tm1a^* allele and genotyping protocol are illustrated in Figure 1A and 1B. The homozygous mutants are viable, but the frequency of homozygous mutant offspring from heterozygous intercross matings is 14%, which is below the expected Mendelian ratio (χ^2^ test, *p*<0.001). Both male and female homozygous mutants are infertile, similar to other *Mcph1* mutants reported previously [9], [13]. There was no evidence of retarded growth in *Mcph1^tm1a^* ^/*tm1a*^ mice (data not shown). Reverse transcription PCR was performed to test the effect of the mutation of *Mcph1* on transcription. The homozygous mutants, heterozygous and wild type mice produced bands of expected size and sequencing of the PCR products validated the results. This indicated that there was residual *Mcph1* transcript in *Mcph1^tm1a^* ^/*tm1a*^ mice. Quantitative real-time PCR revealed the residual transcript of *Mcph1* in the homozygous mice is only 1–4% of the level compared to the wild type mice and the residual levels vary in different organs (Figure 1C).

### *Mcph1*-deficient mice have mild to moderate hearing impairment

**Figure 2:**
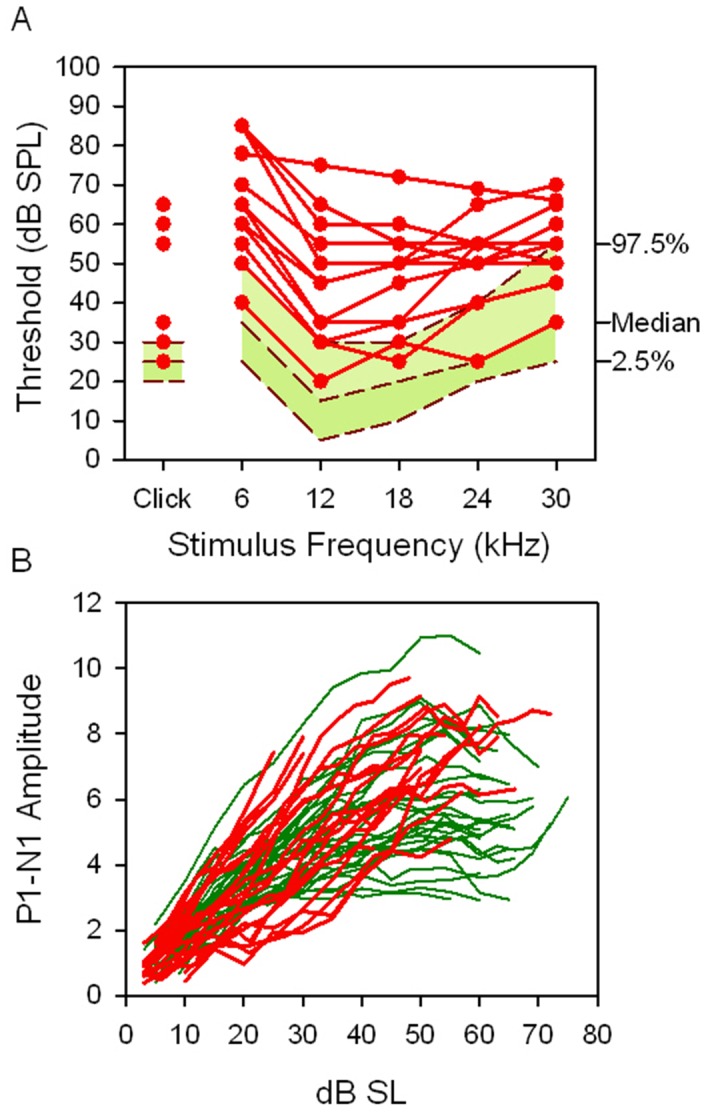
*Mcph1*-deficient mice have mild to moderate hearing impairment characterized with conductive hearing impairment. (A) ABR measurement results of 14 week old *Mcph1^tm1a/tm1a^* mice (n = 11) in MGP showed mild to moderate hearing impairment, or normal hearing compared to control mice. The green baseline area shows a reference range for the control wild type mice with the same genetic background, plotting the median and 2.5 to 97.5 percentile of the population (n = 440). (B) Input-output function (IOF) analysis. The peak-peak amplitude of wave 1 (P1-N1 amplitude) of click-evoked ABRs is plotted as a function of dB SL (Sensation Level, dB above threshold) for wild type (green) and *Mcph1^tm1a/tm1a^* (red) mice. There was no significant difference of IOF slopes of *Mcph1^tm1a/tm1a^* (n = 24, slope  = 0.144+/−0.066; mean +/− SD) and wild type mice (n = 36, slope  = 0.133+/−0.048) (t-test, *p* = 0.444).

**Figure 3:**
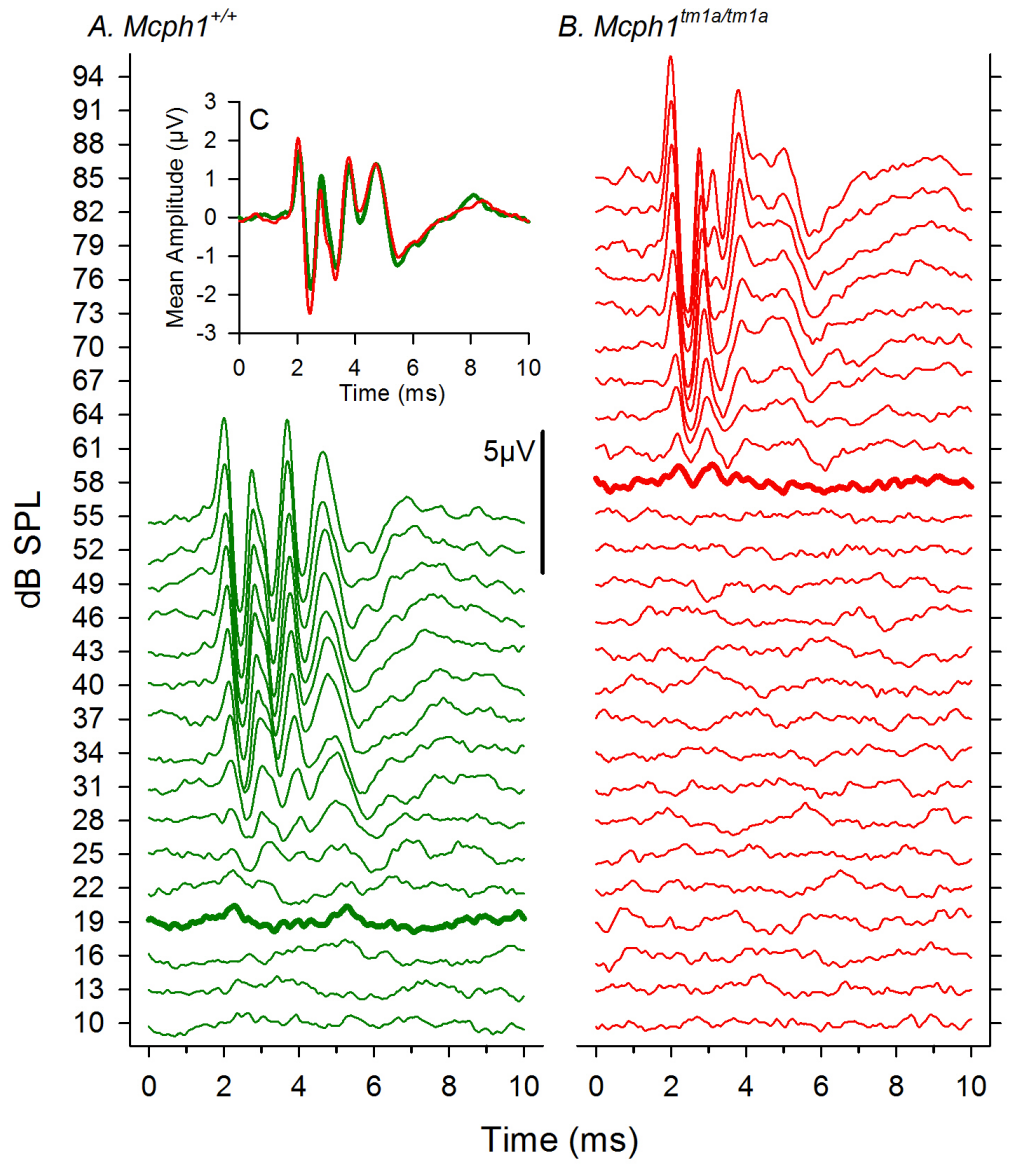
Recurrent ABR measurement indicated the relation between the hearing profile and middle ear defects. (A) Results of recurrent ABR measurement (click thresholds) with age. Hearing impairment can be detected as early as 3 weeks old in *Mcph1^tm1a/tm1a^* mice (n = 13). Hearing profile of the *Mcph1^tm1a/tm1a^* mice showed either a stable, progressive, or fluctuating pattern with age (three of them marked dark). All the wild type (n = 13) and heterozygous (n = 17) mice displayed normal click thresholds with age. (B) Auditory chain (incus-stapes joint) and oval window sound transduction was severely impeded. Normal incus-stapes joint of auditory chain in a *Mcph1^+^* ^/*+*^ mouse, and a clear oval window is necessary for sound vibration conduction. After removing some of the amorphous material in the middle ear cavity of a *Mcph1^tm1a^* ^/*tm1a*^ mouse, the incus-stapes joint (arrow head) and the oval window (arrow) is present but embedded in the amorphous material. Scale bar, 1 mm. (C–F) Correlation between middle ear defects and hearing sensitivity change with time. (C) Normal ABR thresholds and middle ear structure in a wild type mouse: normal middle ear cavity is full of air, tympanic membrane is transparent and normal morphology of ossicles. (D) Progressively elevated ABR thresholds with age in a *Mcph1^tm1a^* ^/*tm1a*^ mouse. Amorphous mass filled the middle ear cavity and outgrew into external ear canal. Ossicles were embedded in the amorphous mass and appeared to have thinner bony structure. (E) Fluctuating ABR thresholds in a *Mcph1^tm1a^* ^/*tm1a*^ mouse. Watery effusion with bubbles was seen in the middle ear cavity and normal gross morphology of ossicles. (F) Stable and moderate hearing impairment in a *Mcph1^tm1a^* ^/*tm1a*^ mouse. The middle cavity was filled with pus-like secretion. Normal gross morphology of ossicles but with rough surface. Scale bar, 1 mm.

Hearing impairment was discovered by ABR measurement in 14 week old *Mcph1^tm1a^* ^/*tm1a*^ mice as part of the standard MGP phenotypic screen (phenotyping overview is available from http://www.sanger.ac.uk/mouseportal/). *Mcph1^tm1a^* ^/*tm1a*^ mice showed mild to moderate hearing impairment with increases of 10–50 decibels (dB) compared to the normal thresholds for both click and pure tone stimuli (6–30 kHz) (Figure 2A). To further characterise the hearing ability of *Mcph1^tm1a^* ^/*tm1a*^ mice and to ask whether the hearing impairment is progressive with age, recurrent ABR measurements were performed in *Mcph1^tm1a^* ^/*tm1a*^, *Mcph1^+^* ^/*tm1a*^ and *Mcph1^+^* ^/*+*^ littermates from 3 weeks to 24 weeks at 3-week intervals. *Mcph1^+^* ^/*tm1a*^ (n = 17) and *Mcph1^+^* ^/*+*^ (n = 13) mice showed normal ABR thresholds, whereas elevated thresholds can be detected in *Mcph1^tm1a^* ^/*tm1a*^ mice as early as 3 weeks of age (n = 13). Thresholds were generally stable over time, although there was progressive or fluctuating hearing impairment over time in some mice (Figure 3A). Heterozygous mice were found to have normal hearing, in accordance with the recessive model of inheritance for patients with *MCPH1* mutations (Figure 3A). *Mcph1^tm1a^* ^/*tm1a*^ mice behaved normally suggesting normal vestibular function. We defined three out of six stimuli tested (click and pure tone stimuli) above the normal hearing reference range as affected in this study, and the penetrance of hearing impairment in *Mcph1^tm1a^* ^/*tm1a*^ mice is around 70% based on this criterion.

**Figure 4:**
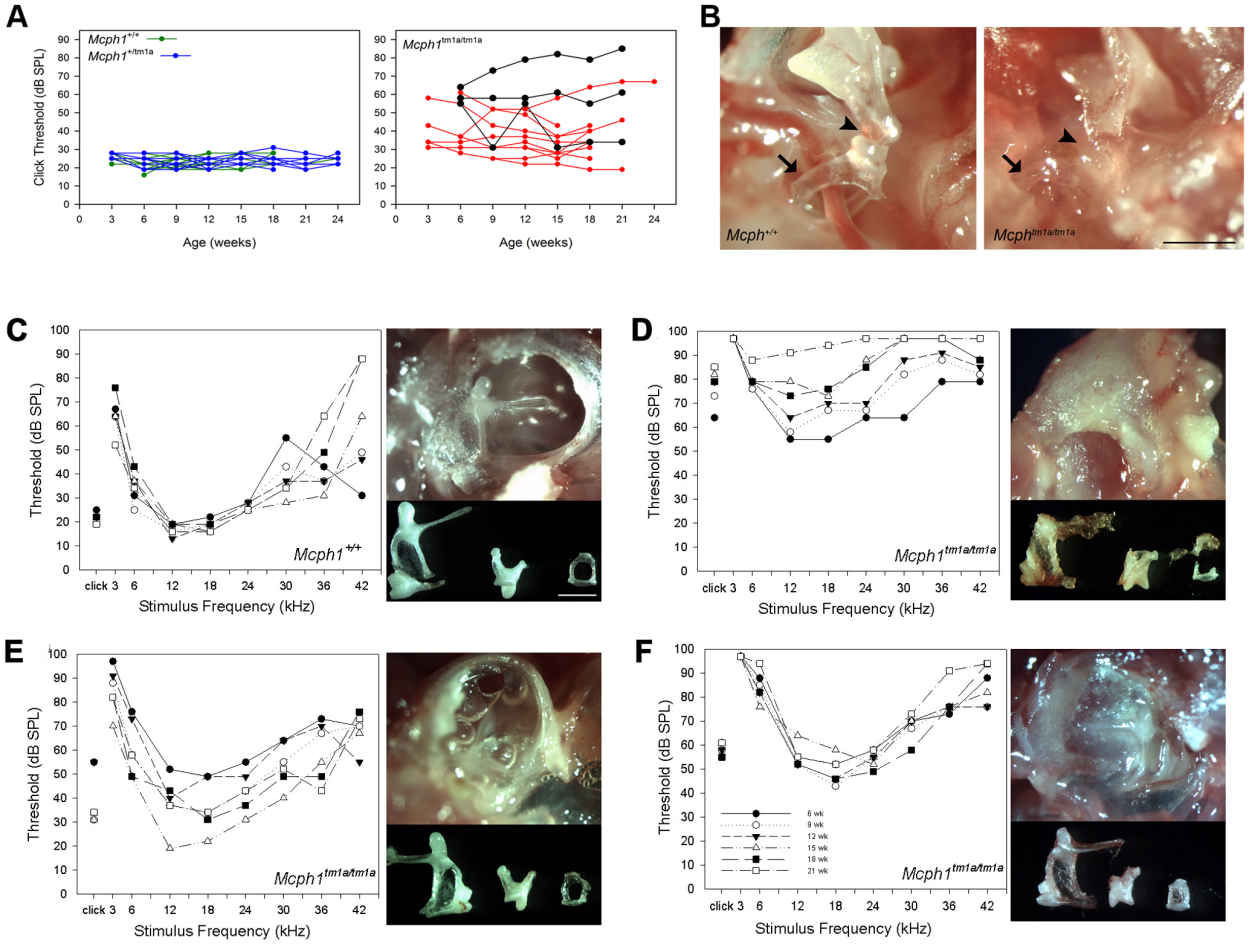
Similar ABR waveforms in *Mcph1^tm1a/tm1a^* and wild type mice at 9 weeks old. A & B illustrate click-evoked ABR waveforms recorded in a wildtype (green) and a*Mcph1^tm1a^* ^/*tm1a*^ (red) mouse, respectively. The dB SPL of the click stimulus for each response is indicated on the abscissa. The scale bar indicates 5 µV amplitude of response. The heavy lines indicate the click-ABR threshold allocated to each mouse. C. (inset) Mean click-evoked ABR waveforms recorded at 21 dB sensation level for wildtype (n = 8, green) and *Mcph1^tm1a^* ^/*tm1a*^ (n = 8, red).

Input-output function analysis showed that growth of amplitude of wave 1 of the click-evoked ABR with increasing sound level above threshold appeared similar in wild type and *Mcph1^tm1a/tm1a^* mice (Figure 2B). ABR waveforms were comparable in shape in mutants compared with controls (Figure 4).

### Anatomical and histological analysis indicates otitis media

To assess the causes of hearing impairment in the *Mcph1^tm1a^* ^/*tm1a*^ mice, anatomical analysis of the middle ear was performed after the completion of the recurrent ABR measurements. All the *Mcph1^+^* ^/*tm1a*^ (n = 17) and *Mcph1^+^* ^/*+*^ (n = 13) mice showed a transparent tympanic membrane, air-filled middle ear cavity, and normal morphology of ossicles (apart from one heterozygous mouse that had some white secretion in the hypotympanum in the right middle ear cavity). Dissection of *Mcph1^tm1a^* ^/*tm1a*^ mice (n = 13) that had elevated ABR thresholds revealed a range of defects in the middle ear including thickened and white bone forming the bulla instead of the normal thin and transparent bone, retracted tympanic membrane, bubbles present underneath the tympanic membrane, or middle ear cavities filled with clear or cloudy fluid. In addition, two *Mcph1^tm1a^* ^/*tm1a*^ mice had an amorphous tissue mass in the middle ear cavity (Figure 3D). The ossicles displayed normal gross morphology, but the mice that had pus-like effusions or amorphous material in the middle ear had a rough surface of the ossicles. However, we did not see bony outgrowths, otorrhea or perforation of the tympanic membrane, which is different from some reported OM mouse models [21]. The correlation between the middle ear effusion and recurrent ABR measurement results is noticeable (Figure 3C–F). Watery secretion with bubbles in the middle ear cavity was seen in the mice that displayed fluctuating ABR thresholds. One mouse that had a pus-like secretion within the middle ear demonstrated relatively stable raised ABR thresholds through all the recurrent ABR measurements. Progressive hearing loss was observed in the mice that had amorphous material in the middle ear cavity, in which we found that the auditory ossicular chain was severely impeded (Figure 3B).

**Figure 5:**
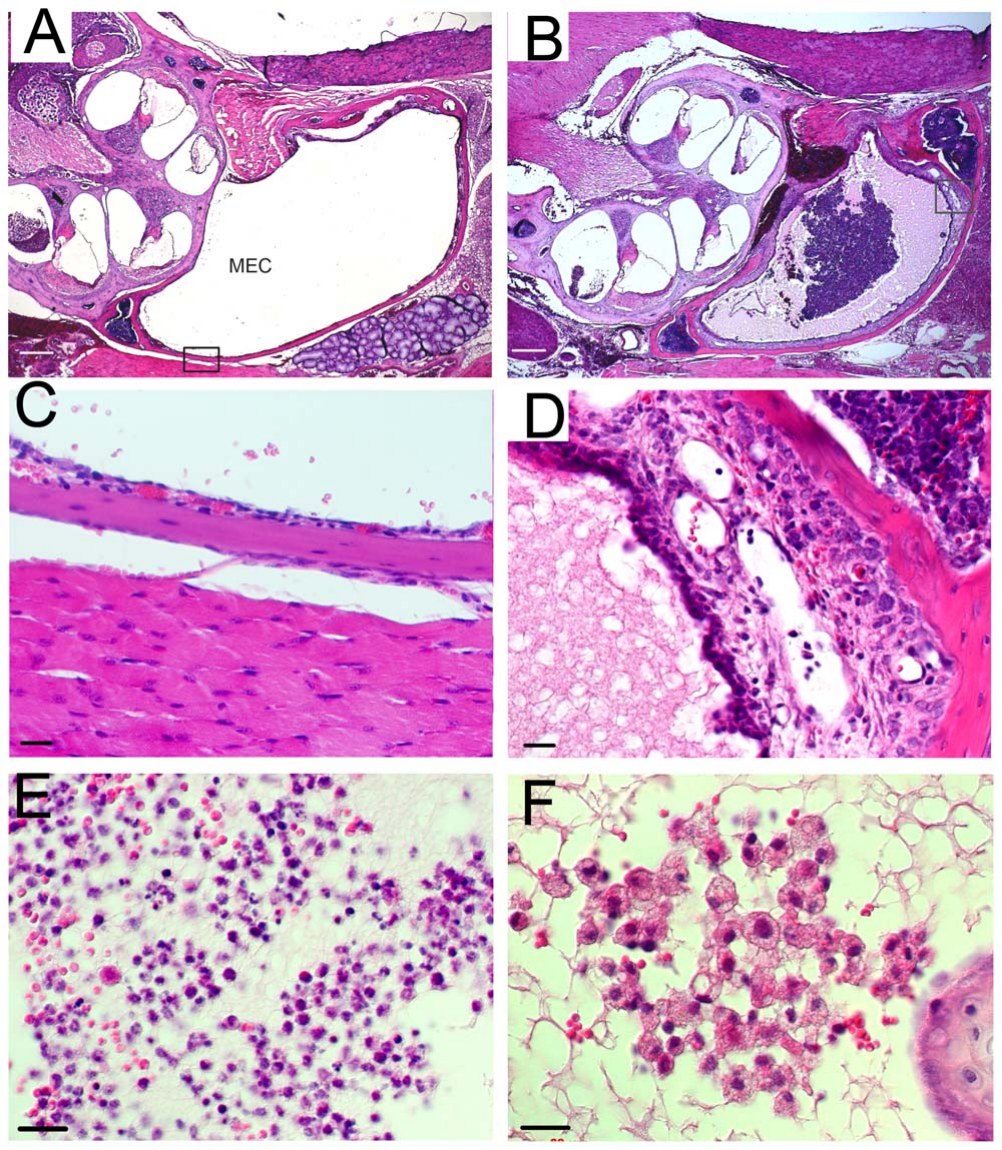
Hematoxylin and eosin staining of the middle ear in adult mice indicated otitis media. Clear middle ear cavity (MEC) and thin mucoperiosteum in wild type mice (A,C). MECs of *Mcph1^tm1a^* ^/*tm1a*^ mice (B,D) were filled with exudate and lined with thickened mucoperiosteum. High magnification for mucoperiosteum of MEC framed in A and B (C,D). Inflammatory cells (E,F) in MECs. Scale bar, 200 µm (A,B), 20 µm (C–F).

Histological examination was performed to investigate the pathological change (Figure 5). Hearing was evaluated by ABR measurement before sectioning the temporal bone. The middle ear cavities of *Mcph1^+^* ^/*tm1a*^ and *Mcph1^+^* ^/*+*^ mice were clear and lined with thin mucoperiosteum. The middle ear cavities of the hearing-impaired *Mcph1^tm1a^* ^/*tm1a*^ mice were filled with exudate and lined with a thickened mucoperiosteum, and occasionally formation of fibrous polyps of mucoperiosteum stroma, indicative of otitis media. The middle ear effusions included a variable amount of inflammatory cells in different mice (mainly granulocytes and scattered foamy macrophages according to the morphology in the haematoxylin/eosin-stained slides). The effusion was confined within the middle ear cavity and did not appear to extend through the round window. In two out of three 60-week old *Mcph1^tm1a^* ^/*tm1a*^ mice, we saw only a few inflammatory cells scattered in the middle ear cavity, but the thickened mucoperiosteum was still present (data not shown). The Eustachian tube had a similar appearance in mutant (n = 4) and control (n = 2 heterozygotes, 2 wild types) mice in serial sections through the tube as it exits the middle ear (data not shown).

We collected tissue from the nasopharynx, middle ears and external ear canal of *Mcph1^tm1a^* ^/*tm1a*^ and wild type mice. DNA was extracted from these and the 16S rRNA gene was amplified via PCR with universal bacterial primers (7f and 1510r). From this, 16S rRNA clone libraries were created for a *Mcph1^tm1a^* ^/*tm1a*^ and wild type mouse for each tissue sampled. The predominant phylotype found to be present in the nasopharynx of both mutant and wild type mice was matched through BLAST analysis to a previously-uncultured *Streptococcus* sequence (ERD01G accession number GQ456229.1) [22]; however, it was also present in the middle ear of the mutant *Mcph1^tm1a^* ^/*tm1a*^ mouse. We were then able to culture this bacterium from the mutant middle ear by plating it onto a variety of media under micro-aerophilic conditions, thus confirming its presence within the tissue. The identity of this isolate as *Streptococcus* bacterium (Strep ERD01G) was confirmed by 16S rRNA PCR (Derek Pickard, Trevor Lawley and Mark Stares, personal communication).

### Normal inner ear structure

**Figure 6:**
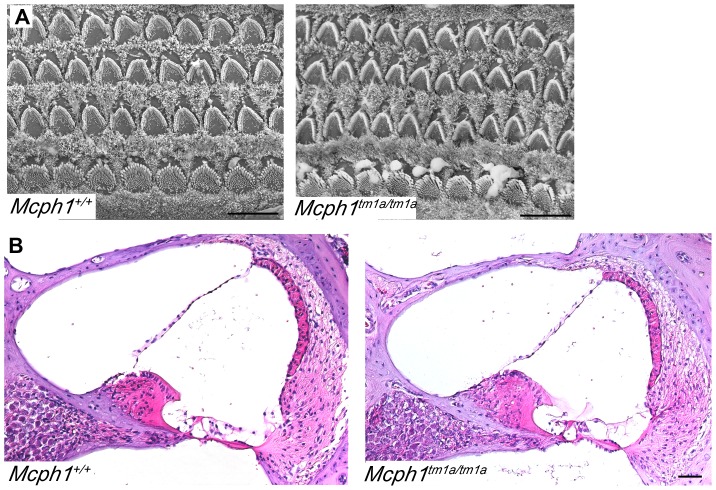
Normal inner ear structure in *Mcph1^tm1a/tm1a^* mice. (A) Scanning electron microscope (SEM) showed normal development of hair cells at P4 in *Mcph1^tm1a^* ^/*tm1a*^ mice (*Mcph1^+^* ^/*+*^, n = 3; *Mcph1^tm1a^* ^/*tm1a*^, n = 3. scale bar, 10 µm). (B) HE slides displayed comparable structure of inner ears (basal turn) in *Mcph1^+^* ^/*+*^ and *Mcph1^tm1a^* ^/*tm1a*^ mice at 4–5 weeks old (*Mcph1^+^* ^/*+*^, n = 3; *Mcph1^tm1a^* ^/*tm1a*^, n = 3. scale bar, 50 µm).

To learn whether *Mcph1^tm1a^* ^/*tm1a*^ mice have inner ear defects, scanning electron microscopy (SEM) and temporal bone sections were used to examine cochleae in young pups and adult mice respectively. At postnatal day 4, *Mcph1^tm1a^* ^/*tm1a*^ mice showed normal morphology of the upper surface of the organ of Corti by SEM (Figure 6A). Adult *Mcph1^tm1a^* ^/*tm1a*^ mice showed a normal gross anatomy of the inner ear (data not shown) and there was no evidence of any abnormality of the cochlea in *Mcph1^tm1a^* ^/*tm1a*^ mice (4–5 week old, Figure 6B).

### Expression of Mcph1 in the middle ear

**Figure 7:**
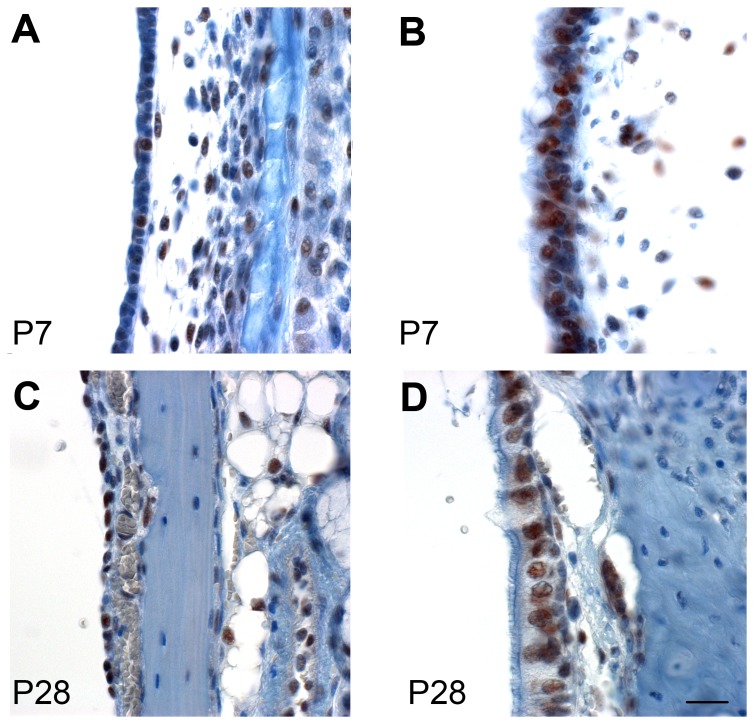
Mcph1 is expressed in the middle ear. Immunochemistry using an antibody shows that Mcph1 (brown labelling) is expressed in epithelial cells of the middle ear cavity at P7 (A,B) and P28 (C,D) wild type mice. Mcph1 is expressed in both ciliated (B,D) cells close to orifice of Eustachian tube and non-ciliated cells (A,C). Scale bar, 20 µm.

Expression of Mcph1 in epithelial cells of the middle ear cavity (Figure 7) was revealed by immunohistochemistry. Before maturation of the middle ear (postnatal day 7), Mcph1 was expressed in the epithelial cells of the middle ear cavity and appeared stronger in the ciliated cells than the non-ciliated cells. The expression was seen in some cells of the middle ear mesenchyme as well at P7. In adult mice (4–5 weeks old), the expression appeared stronger than that of P7 mice and was present and appeared similar in both ciliated and non-ciliated cells.

### Smaller skull size with normal skull structure

**Figure 8:**
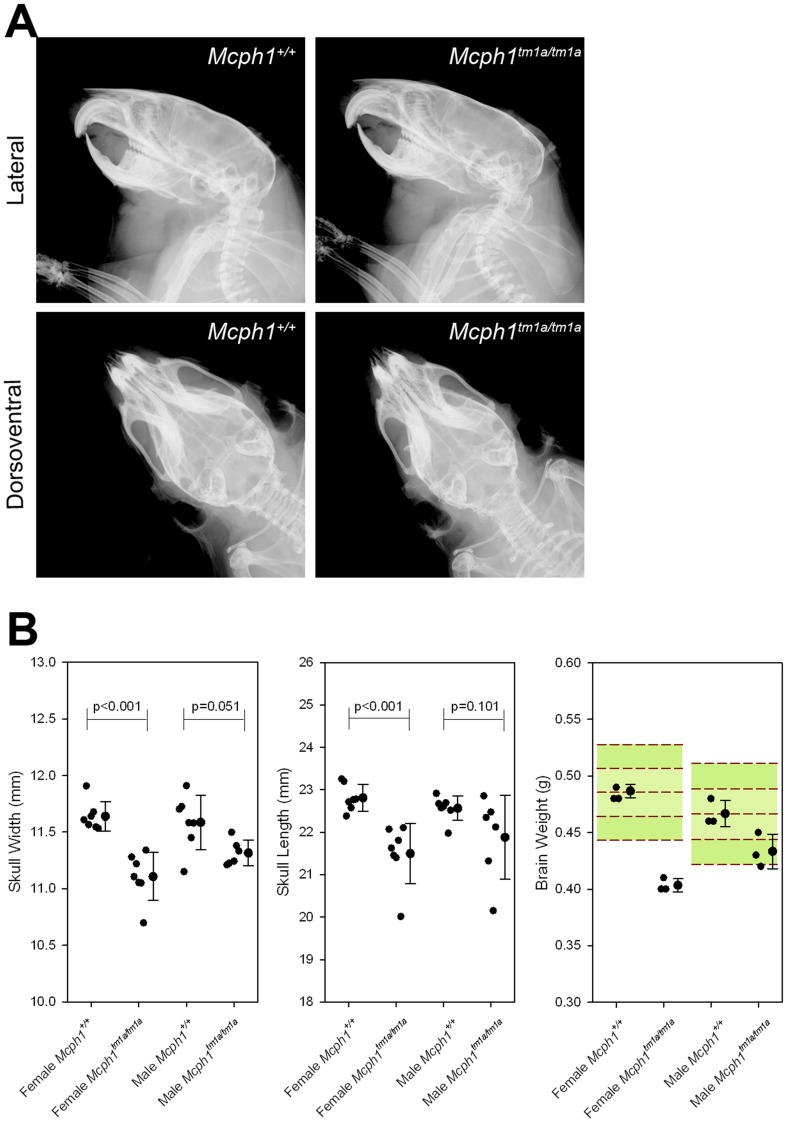
*Mcph1^tm1a^* ^/*tm1a*^ mice have normal skull structure but smaller skull size. (A) X-ray assay showed comparable structure of craniofacial skeleton between wild type and *Mcph1^tm1a^* ^/*tm1a*^ mice at 14 weeks old. (B) Measurement showed that skull width and length in female *Mcph1^tm1a^* ^/*tm1a*^ mice (n = 6) are significantly smaller than those of wild type mice (n = 7) (Rank Sum test). The weight of the brain was measured at 16 weeks of age showed that female and male *Mcph1^tm1a^* ^/*tm1a*^ mice (n = 3, each sex) had lighter brain than the local control mice (n = 3, each sex) and MGP wild type mouse baseline (female, n = 10; male n = 187).

We examined the skeleton of the *Mcph1^tm1a^* ^/*tm1a*^ mice by X-ray at 1eeks of age. *Mcph1^tm1a^* ^/*tm1a*^ mice showed a similar skull structure compared with the wild type mice (Figure 8A). However, skull width and length of female *Mcph1^tm1a^* ^/*tm1a*^ mice were significantly smaller than those of wild type mice (Figure 8B). The weight of the brain was measured at 16 weeks old, and both male and female *Mcph1^tm1a^* ^/*tm1a*^ mice had lighter brain weight than wild type mice.

### Increased prevalence of micronucleated normochromatic erythrocytes

**Figure 9:**
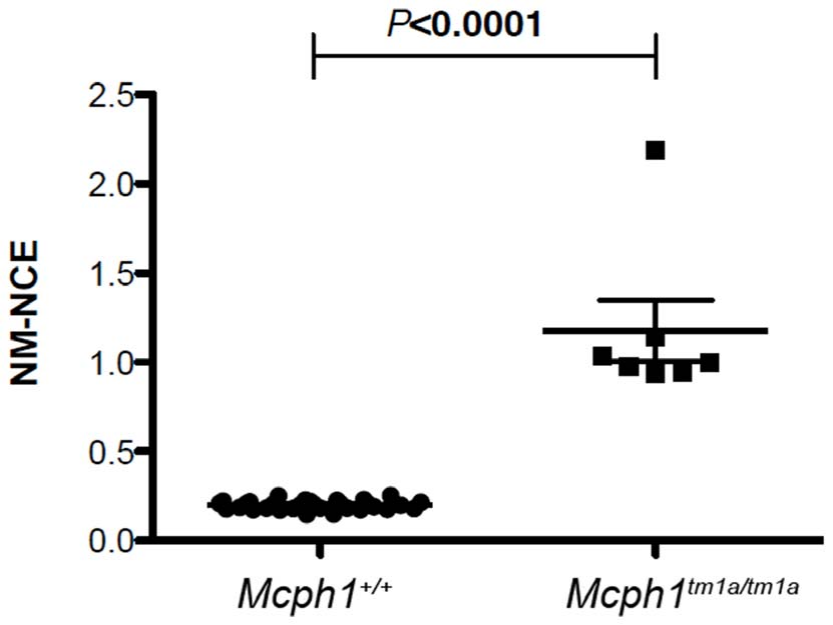
*Mcph1^tm1a/tm1a^* mice show evidence of genomic instability. Adult *Mcph1^tm1a^* ^/*tm1a*^ (n = 7) mice showed increased genomic instability when compared to wild type mice (n = 34) as determined by an increased prevalence of micronucleated normochromatic erythrocytes (MN-NCE). Data is presented as the Mean ± SEM.

There was an increased prevalence of micronucleated normochromatic erythrocytes (MN-NCE) in *Mcph1^tm1a^* ^/*tm1a*^ mice compared to the control mice (Figure 9) indicating increased genomic instability.

### Normal control of *Salmonella* Typhimurium and *Citrobacter rodentium* challenge

**Figure 10:**
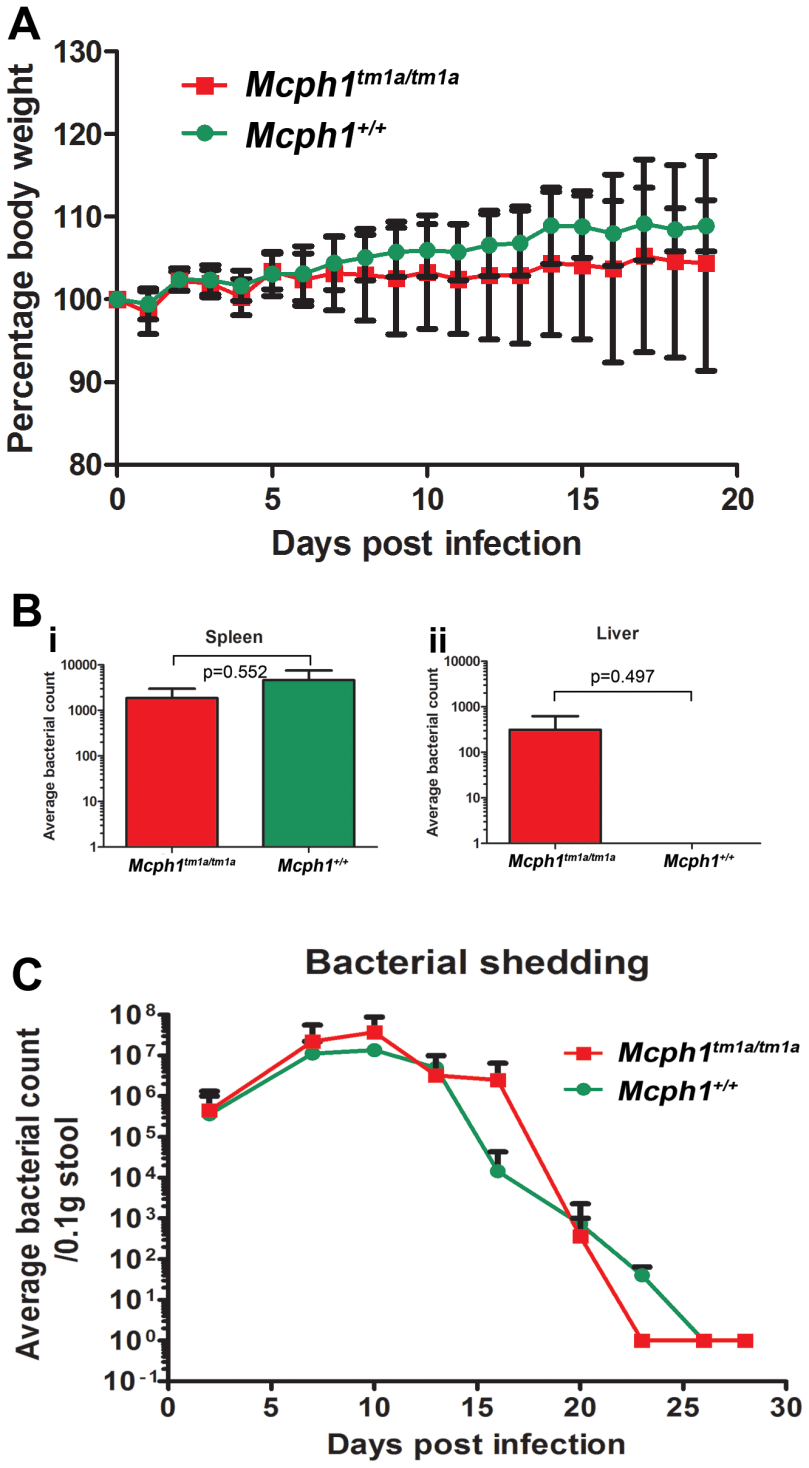
*Mcph1^tm1a/tm1a^* mice had normal control of *Salmonella* Typhimurium and *Citrobacter rodentium* challenge. (A) No difference of weight change (Mean ± SEM) between *Mcph1^tm1a/tm1a^* (n = 4) and *Mchp1^+/+^* (n = 8) mice infected by *Salmonella* Typhimurium and monitored by weight loss for 21 days. (B) Viable bacterial counts from the spleen (i) and the liver (ii) from the same mice infected by *Salmonella* Typhimurium at 21 days post infection. There was only one *Mcph1^tm1a/tm1a^* mouse out of 4 tested with any bacteria present in the liver. Mann Whitney U tests were used, p (two-tailed) is indicated in the figure. (C) Viable bacterial counts (Mean ± SEM) being shed in the stool from *Mcph1^tm1a/tm1a^* (n = 5) and *Mchp1^+/+^* (n = 5) mice were similar over a 28 day infection of *Citrobacter rodentium*.

Following a systemic *Salmonella* Typhimurium challenge, *Mcph1^tm1a^* ^/*tm1a*^ mice had a similar change of body weight compared to control mice over a 21 day period following the infection (Figure 10A). Counts of bacteria in the livers and spleens at day 21 were no different in *Mcph1^tm1a^* ^/*tm1a*^ mice compared to control mice and the mice cleared the bacteria from the system (Figure 10B). When challenged with the gastric pathogen *Citrobacter rodentium,* there was no difference between *Mcph1^tm1a^* ^/*tm1a*^ and control mice in colonisation and bacterial shedding in the stool of infected mice (Figure 10C).

### Increased B cells but normal antibody production

**Figure 11:**
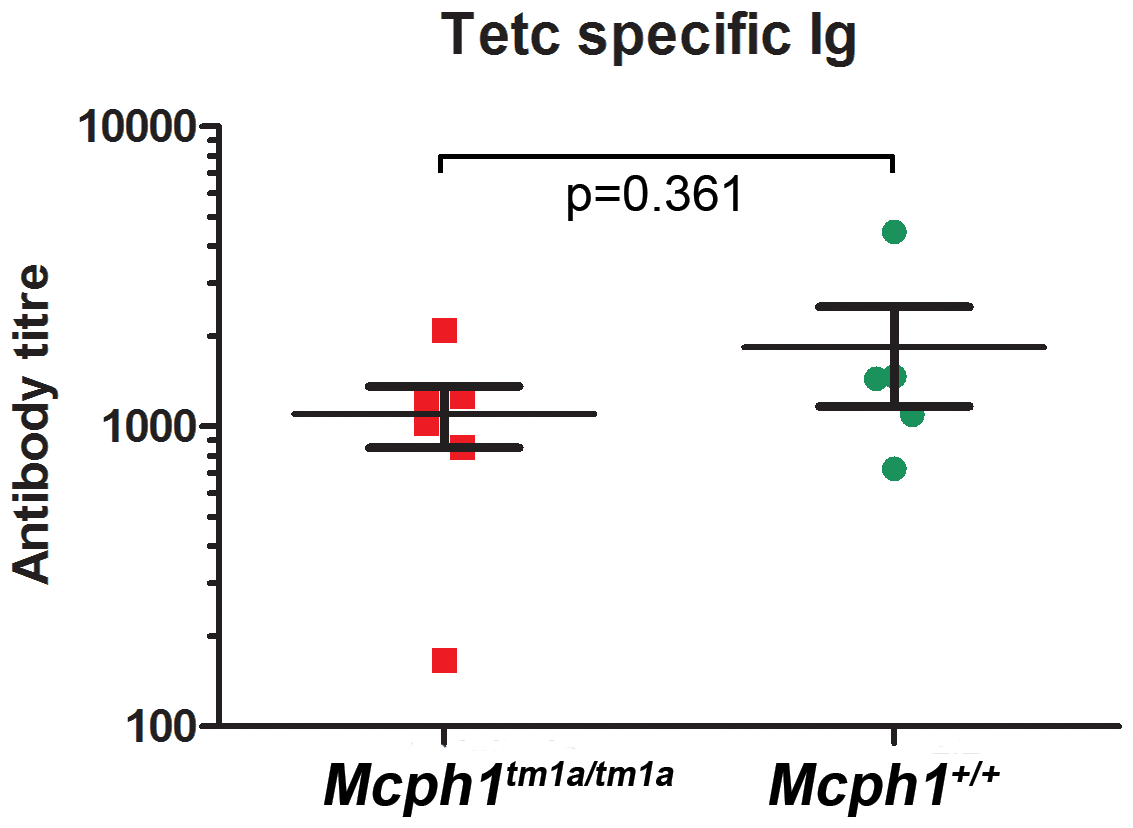
Measurement of anti-TetC specific antibodies in immunised mouse serum. *Mcph1^tm1a/tm1a^* and *Mchp1^+/+^* mice were immunised intranasally with fragment C of tetanus toxin on day 0, 7 and 21. Presence of antigen-specific Ig in serum isolated at day 28 was showed no difference in *Mcph1^tm1a/tm1a^* (n = 6) and *Mchp1^+/+^* (n = 5). The solid bar represents the Mean ± SEM. Mann Whitney U tests were used, p (two-tailed) is indicated in the figure.

There was no evidence of any immune defects or activation after analysis of the peripheral blood leukocytes by flow cytometry (data not shown). The only significant difference was an increase in the circulating B cell frequency in female *Mcph1^tm1a^* ^/*tm1a*^ mice, however there was no difference in the maturity of these B cells as determined by cell surface IgD expression (data not shown). To test if this alteration to the B cell frequency resulted in any changes in antibody production, we performed a prime boost immunisation with Fragment C of tetanus toxin. However, in agreement with the normal response to infection there was no significant change in antibody level in the *Mcph1^tm1a^* ^/*tm1a*^ mice (Figure 11).

### Ocular abnormalities

**Figure 12:**
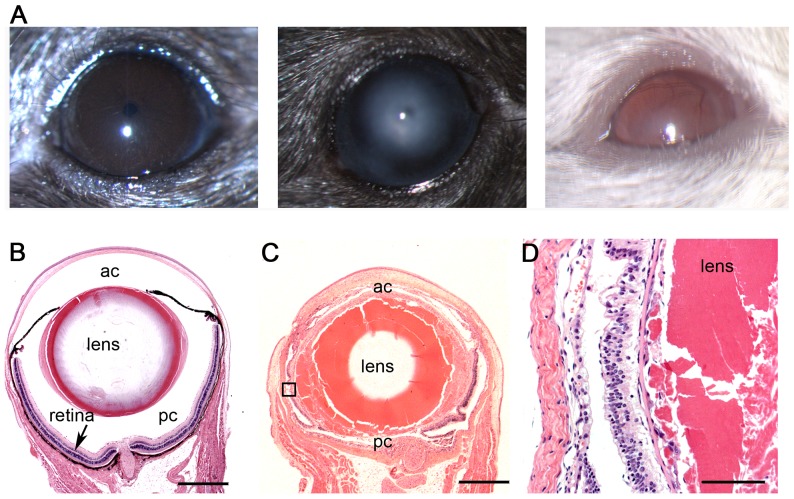
*Mcph1^tm1a^* ^/*tm1a*^ mice have ocular abnormalities. (A) Slit lamp images (12× magnification) revealed corneal (center) opacity and vascularisation (right) in *Mcph1^tm1a^* ^/*tm1a*^ mice. The difference of ocular abnormality portion is significant between wild type (n = 23) and *Mcph1^tm1/tm1a^* (n = 14) mice (Fischer's exact test: p = 0.002). (B) Wild type eye shows normal lens and retina. The anterior and posterior chamber spaces are well defined. ac  =  anterior chamber; pc  =  posterior chamber. (C) *Mcph1^tm1a^* ^/*tm1a*^ eye shows cataractous lens and thin retina. The anterior and posterior chambers are collapsed. Scale bar, 500 µm (B,C). (D) Inset from (C) with solid line shows cataractous lens and loss of retinal cell layers. Scale bar, 100 µm.

A significant proportion of *Mcph1^tm1a^* ^/*tm1a*^ mice displayed ocular abnormalities including corneal opacity and vascularisation (Figure 12A). Abnormal histopathology of *Mcph1^tm1a^* ^/*tm1a*^ mice was revealed by sections in the pupil-optic nerve plane including collapsed anterior and posterior chambers, cataracts of lens, and disorganized and degenerated retinal layers (Figure 12B).

### Other screening tests

*Mcph1^tm1a^* ^/*tm1a*^ mice did not show any abnormalities in other screening tests such as haematology of peripheral blood, clinical chemistry or intraperitoneal glucose tolerance tests (for more details refer to http://www.sanger.ac.uk/mouseportal/).

## Discussion

We report here a new mouse with a targeted mutation of *Mcph1* (*Mcph1^tm1a(EUCOMM)Wtsi^*, knockout-first design [5]) resulting in a severe reduction in transcript level of *Mcph1* in the homozygous mutant mice to less than 4% of the wild type level. During the standardised phenotypic screen of these mutants, we found an unexpected phenotype: mild to moderate hearing impairment. ABR thresholds were raised uniformly across all frequencies tested and the growth of amplitude of the waveform with increasing sound stimulation above threshold was similar in mutants and controls, both features consistent with a conductive hearing impairment. Subsequent ABR measurement, dissection of the middle ear and histopathology indicated otitis media with effusion was present to varying degrees in the mutant middle ears. We found expression of Mcph1 in the epithelia lining the middle ear consistent with its involvement in otitis media. These findings suggest that *Mcph1^tm1a^* ^/*tm1a*^ mice are a model for one form of heritable otitis media and reveal a new molecule involved in the pathogenic pathways underlying otitis media that can be used to unravel the underlying mechanisms irrespective of the initial trigger.

ABR has been used extensively for assessment of mouse auditory function and can detect moderately-raised thresholds due to middle ear inflammation among many other mechanisms underlying hearing impairment [23]. Compared to using the clickbox, which produces an intense auditory stimulus and can only be used to search for deaf or severely hearing-impaired mice by lack of a Preyer reflex (ear flick), ABR measurement has the advantage of detecting mild to moderate hearing loss [24], which is the expected threshold elevation in conductive hearing impairment. ABR is efficient for detecting mild alteration of hearing function, although some mice with OM assessed by histology may have normal ABR thresholds if the effusion accumulated in the middle ear is not enough to interfere with normal sound transmission. All the mice in this study were bred in a specific pathogen-free animal facility and had a penetrance of hearing impairment around 70%, but there may be a higher penetrance if the mice are bred in a conventional environment.

Previous studies showed that the inner ear might be involved in long-term hearing threshold elevations in OM [25], [26] and the round window was proposed as the most likely site of entry of inflammatory mediators and noxious substances [27]–[30]. However, we did not observe obvious degeneration of the spiral ganglion, hair cells or lateral wall, or serous labyrithitis in sections of hearing-impaired *Mcph1^tm1a^* ^/*tm1a*^ mice. Furthermore, round windows appeared intact and the exudate was confined within the middle ear cavity. Most *Mcph1^tm1a^* ^/*tm1a*^ mice showed ABR threshold elevations no higher than 40 dB, which is what is expected in conductive hearing impairment and all tested frequencies were affected which is another characteristic of conductive hearing impairment. Furthermore, input-output function analysis suggested that there is no evidence for a recruitment-type effect in the hearing-impaired *Mcph1^tm1a^* ^/*tm1a*^ mice, where a steeper slope would be seen and be indicative of recruitment of additional auditory nerve fibers into the ABR wave 1 component with increasing sound levels as a result of sensorineural hearing loss. Thus, the features of hearing impairment seen in the *Mcph1^tm1a^* ^/*tm1a*^ mice are consistent with conductive impairment. We noticed a few *Mcph1^tm1a^* ^/*tm1a*^ mice had ABR threshold elevation higher than 40 dB (Figure 4D), so there may be some sensorineural component involved, in which case, the noxious substances may permeate through an intact round window membrane [31].

The Eustachian tube is a tube that links the nasopharynx to the middle ear and plays a critical role in the development of OM due to its functions in pressure equalization and mucus drainage and it is thought to be the main route of infection into the middle ear. Eustachian tube dysfunction can be caused by developmental anatomical anomalies [32], [33], adenoid [34] or tumor [35] blockage in the nasopharynx, or cilial defects in the Eustachian tube [36], [37]. We observed that some *Mcph1^tm1a^* ^/*tm1a*^ mice had fluctuating ABR thresholds, and one possible explanation for this may be that the accumulated effusion in middle ear cavities can be drained through the Eustachian tube, through which hearing can be restored partially. However, we saw no obvious abnormality in the Eustachian tube of mutants in serial sections.

The clinical classification of OM remains a topic of disagreement [38]. One classification divides cases into acute OM, OM with effusion (OME), chronic suppurative OM, and adhesive OM. In this study, we did not see typical signs of acute OM (red eardrum) or chronic suppurative OM (perforated eardrum and discharge/otorrhoea). OME is the most common cause of hearing impairment in children in the developed world [39] and a prospective twin study suggested that genetics played a large role in OME [1]. In our study of *Mcph1^tm1a^* mutants, dissection of the middle ears revealed effusion or amorphous mass which appeared more like the signs of OM with effusion or adhesive OM. So we suggest that *Mcph1^tm1a^* ^/*tm1a*^ mouse is a suitable mouse model for research into human OME and adhesive OM. There are no signs of multi-systemic inflammation or any indications of immunodeficiency in *Mcph1^tm1a^* ^/*tm1a*^ mice indicating normal overt immune function. The only difference was the increased B cell percentage in the blood of female mutants. This observation in naïve animals is further supported by the ability of these mice to control their response to *Salmonella* and *Citrobacter*, which are pathogens that challenge the immune system both systemically and at a mucosal surface. Furthermore, antibody production after immunisation was the same in *Mcph1^tm1a^* ^/*tm1a*^ and control mice. Overall these findings suggest that *Mcph1^tm1a^* ^/*tm1a*^ are not immunocomprised and do not display any indications of autoimmunity or inflammation.

MCPH1 protein is ubiquitously expressed [40]. When we examined the expression of Mcph1 in the middle ear, we found Mcph1 was expressed in middle ear mucosal cells and was especially marked in 4–5 week old adults. Four-five weeks old is within the time of occurrence of OM. The major part of the middle ear is lined with simple, non-keratinizing squamous cells with or without microvilli. Ciliated and secretory cells are concentrated around the Eustachian tube orifice [41]. Non-ciliated secretory cells including goblet cells, completely filled with secretory granules, that produce the mucin [42] and ciliated cells are important for the clearance of the mucus. The expression of *Mcph1* in both non-ciliated and ciliated cells is consistent with a role for Mcph1 in the production and clearance of middle ear mucus.

Mcph1 localises to centrosomes [13], [43] suggesting that it may affect the primary cilium. OM in *Mcph1^tm1a^* ^/*tm1a*^ mice may be due to a cilium dysfunction causing reduced clearance leading to accumulation of mucin. Disorganized and degenerated retinal layers in eyes, where photoreceptors have an integral cilium, and male infertility also may be explained by any cilium defects. These hypotheses need future investigation. However, *Mcph1^tm1a^* ^/*tm1a*^ mice did not display phenotypes normally associated with ciliopathies, such as situs inversus or renal cystic disease, suggesting that sufficient amounts of Mcph1 are available in the mutant for functional cilia formation in the majority of cells.

Very recently, three different *Mcph1*-deficient mouse models have been reported [9], [13], [44]. *Mcph1^tm1a/tm1a^* mice in our study had some similarities with the reported mouse models such as reduced birth rate and infertility in both sexes. Increased genomic instability is another common phenotype shared by all the *Mcph1*-deficient mouse models. One of these three mutants clearly exhibited small brains, mimicking microcephaly in humans [13]. The lack of microcephaly in one mouse model was associated with a hypomorphic mutation generated by inserting a gene-trap cassette into intron 12 [44]. We found that female *Mcph1^tm1a/tm1a^* mice had smaller skull sizes, as observed in *MCPH1* patients. There is a small amount of residual transcript revealed by real-time PCR in *Mcph1^tm1a/tm1a^* mice, suggesting that the lack of a microcephaly phenotype cannot be explained simply by the presence of residual Mcph1 mRNA or protein. Lymphoblastoid cell lines carrying a *MCPH1* patient mutation C74G (S25X) also suggest a more complex explanation, as these cells expressed residual MCPH1 protein but were derived from a patient with microcephaly [11].

OM or hearing impairment has not been reported in human patients or mouse models with *MCPH1* mutations previously. One possible explanation for this is that hearing impairment can easily be missed in the mouse. Also, owing to practical difficulties [40], OM occurrence in microcephaly patients may be overlooked. As OM has been detected frequently in these mouse mutants, it may be worth looking specifically for OM in patients with microcephaly, as OM can cause long-term problems if untreated.

Besides OM, hearing impairment and smaller brain and skull sizes, we observed other defects in *Mcph1^tm1a/tm1a^* mice. Similar to studies of other *Mcph1* mutants, we found that *Mcph1*-deficient mice have defects in DNA damage repair revealed by the increased prevalence of micronucleated normochromatic erythrocytes. Eye abnormalities revealed by gross morphology and histopathology present to varying degrees in the mutants implicating *Mcph1* function in vision, but have not previously been reported in *MCPH1* patients or mouse models.

*Mcph1* was proposed as a potential tumour suppressor because decreased levels of Mcph1 were detected in several types of human cancer including breast and ovarian cancers [10]. The high level of micronuclei in erythrocytes of *Mcph1^tm1a/tm1a^* mutants suggests genomic instability so is consistent with a role in cancer. However, the four available *Mcph1* mutant mouse lines have not been reported to show any excess of tumours, although none have been systematically aged and examined appropriately to detect tumours. Furthermore, there is anecdotal evidence that the incidence of cancer in *MCPH1* patients is low [40]. The inconsistency between the reduced *MCPH1* expression in human cancer cells and increased micronuclei in the mice reported here on the one hand and the lack of reported tumour development in mouse *Mcph1* mutants and *MCPH1* patients on the other hand may reflect the small numbers of individuals studied appropriately.

The knockout-first allele which *Mcph1^tm1a/tm1a^* mice carry can produce reporter knockouts, conditional knockouts, and null alleles following exposure to site-specific recombinases Flp and Cre [5], so the *Mcph1^tm1a^* ^/*tm1a*^ mouse could provide useful tools for further research to unravel the underlying mechanism of OM. The discovery of a role for *Mcph1* in predisposition to OM expands our knowledge of genetic factors underlying OM. Rapid advances in sequencing technologies have already proved valuable in finding novel OM genes [45]. Undoubtedly, combining mouse models with methods for analysing human populations such as genome wide association studies and massively parallel sequencing will contribute to the long-term goal of the development of preventative and therapeutic approaches for OM.
