# Correction: *Mcph1*-Deficient Mice Reveal a Role for MCPH1 in Otitis Media

**DOI:** 10.1371/annotation/fadb7426-df05-4ec5-a0ba-21981295b0eb

**Published:** 2013-06-19

**Authors:** Jing Chen, Neil Ingham, Simon Clare, Claire Raisen, Valerie E. Vancollie, Ozama Ismail, Rebecca E. McIntyre, Stephen H. Tsang, Vinit B. Mahajan, Gordon Dougan, David J. Adams, Jacqueline K. White, Karen P. Steel

Figures 3 and 4 were incorrectly switched. The locations of the legends for Figures 3 and 4 are correct.

